# Supplementary figures and images for: Neural networks enable efficient and accurate simulation-based inference of evolutionary parameters from adaptation dynamics
Source: PLoS Biol. 2022 May 27;20(5):e3001633. doi: 10.1371/journal.pbio.3001633 (PMC9140244; doi:10.1371/journal.pbio.3001633)

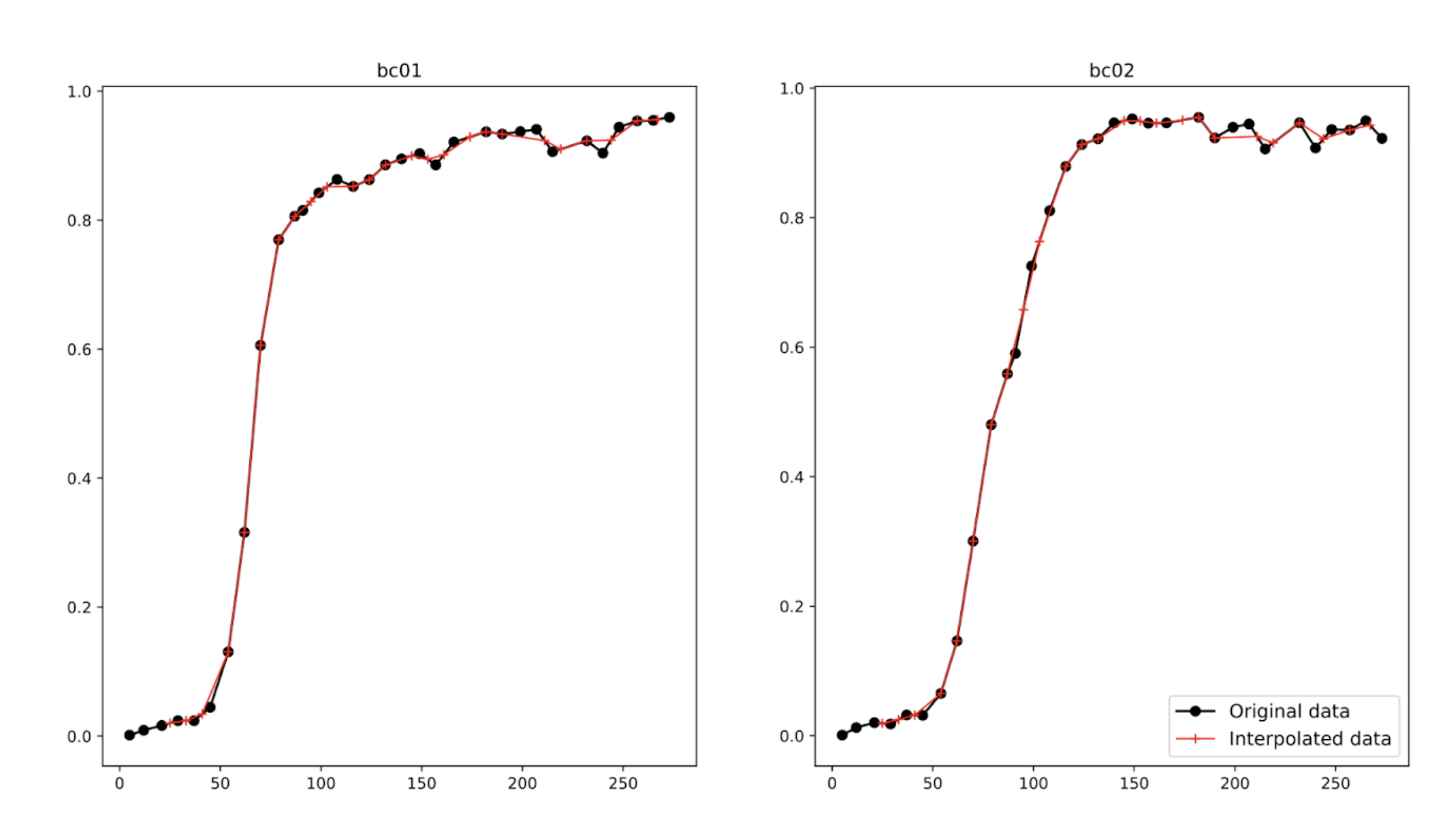

Supplement: S1 Fig — Populations gln01-gln09 and bc01-bc02 have different time points—the gln populations have 25 time points in total, whereas the bc populations have 32 time points in total. Of these, 12 of the time points are the same in both populations. To match the time points in the gln populations, we interpolated from the 2 nearest time points in the bc populations (using pandas.DataFrame.interpolate(“values”)). This way, we can use the same data (same time points) for inference for all 11 populations so that we can use the same amortized NPE posterior to infer parameters for both gln populations and bc populations. Original bc data are shown as black dots, the matched data, with interpolated time points, is shown as red crosses. Data and code required to generate this figure can be found at https://doi.org/10.17605/OSF.IO/E9D5X. NPE, Neural Posterior Estimation. (PNG) [file pbio.3001633.s003.png]

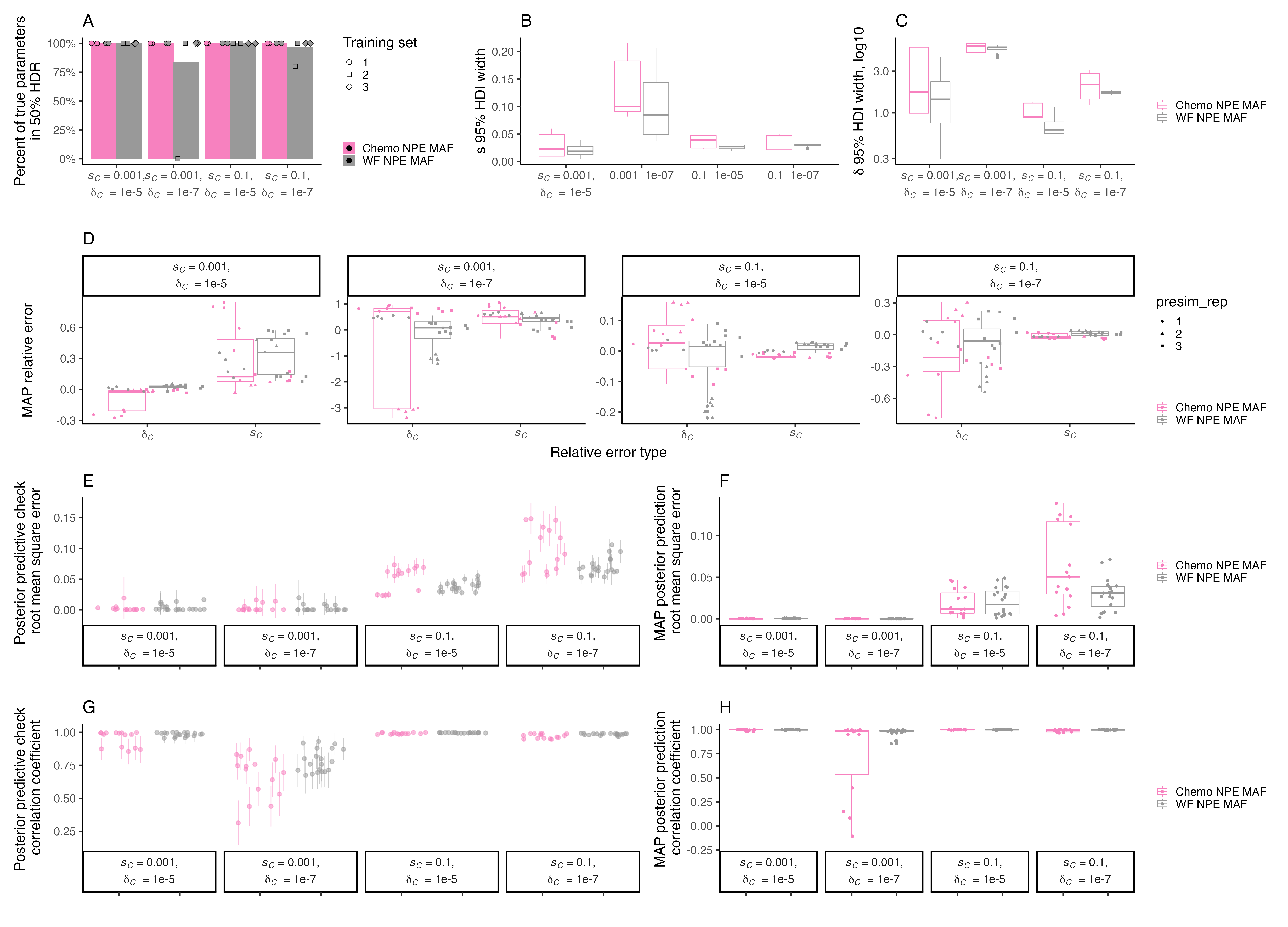

Supplement: S2 Fig — These show the results of inference on 5 simulated synthetic observations generated using either the WF or chemostat (Chemo) model (and inference performed with the same model) per combination of fitness effect sC and formation rate δC. Here, we show the results of performing one training set with NPE with MAF using 100,000 simulations for training and using the same amortized network to infer a posterior for each replicate synthetic observation. (A) Percentage of true parameters within the 50% HDR. (B) Distribution of widths of the fitness effect sC 95% HDI calculated as the difference between the 97.5 percentile and 2.5 percentile, for each inferred posterior distribution. (C) Distribution of the number of orders of magnitude encompassed by the formation rate δC 95% HDI, calculated as difference of the base 10 logarithms of the 97.5 percentile and 2.5 percentile, for each inferred posterior distribution. (D) Log ratio MAP estimate as compared to true parameters for sC and δC. Note that each panel has a different y-axis. (E) Mean and 95% confidence interval for RMSE of 50 posterior predictions as compared to the synthetic observation for which inference was performed. (F) RMSE of posterior prediction generated with MAP parameters as compared to the synthetic observation for which inference was performed. (G) Mean and 95% confidence interval for correlation coefficient of 50 posterior predictions compared to the synthetic observation for which inference was performed. (H) Correlation coefficient of posterior prediction posterior prediction generated with MAP parameters compared to the synthetic observation for which inference was performed. Data and code required to generate this figure can be found at https://doi.org/10.17605/OSF.IO/E9D5X. HDI, highest density interval; HDR, highest density region; MAF, masked autoregressive flow; MAP, maximum a posteriori; NPE, Neural Posterior Estimation; RMSE, root mean square error; WF, Wright–Fisher. (PNG) [file pbio.3001633.s004.png]

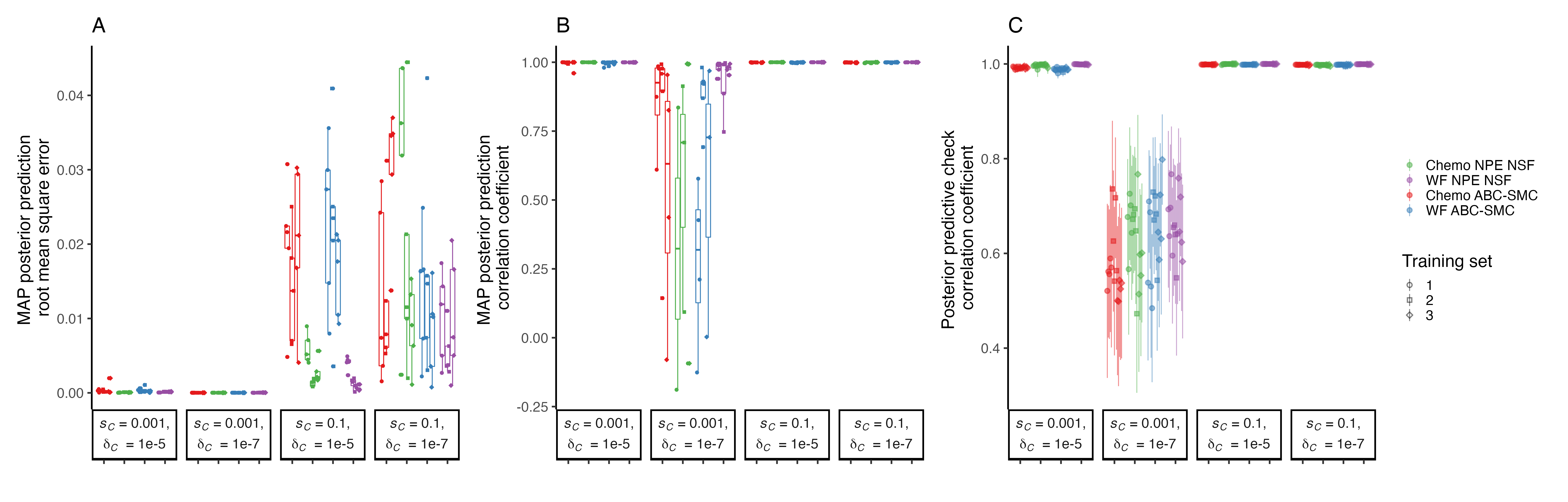

Supplement: S3 Fig — Results of inference on 5 simulated single synthetic observations generated using either the WF or chemostat (Chemo) model (and inference performed with the same model) per combination of fitness effect sC and formation rate δC. Here, we show the results of performing training with NPE with NSF using 100,000 simulations for training and using the same amortized network to infer a posterior for each replicate synthetic observation, or ABC-SMC when the training budget was 10,000. (A) RMSE (lower is better) of posterior prediction generated with MAP parameters as compared to the synthetic observation on which inference was performed. (B) Correlation coefficient (higher is better) of posterior prediction generated with MAP parameters compared to the synthetic observation on which inference was performed. (C) Mean and 95% confidence interval for correlation coefficient (higher is better) of 50 posterior predictions (sampled from the posterior distribution) compared to the synthetic observation on which inference was performed. Data and code required to generate this figure can be found at https://doi.org/10.17605/OSF.IO/E9D5X. ABC-SMC, Approximate Bayesian Computation with Sequential Monte Carlo; MAP, maximum a posteriori; NPE, Neural Posterior Estimation; RMSE, root mean square error; WF, Wright–Fisher. (PNG) [file pbio.3001633.s005.png]

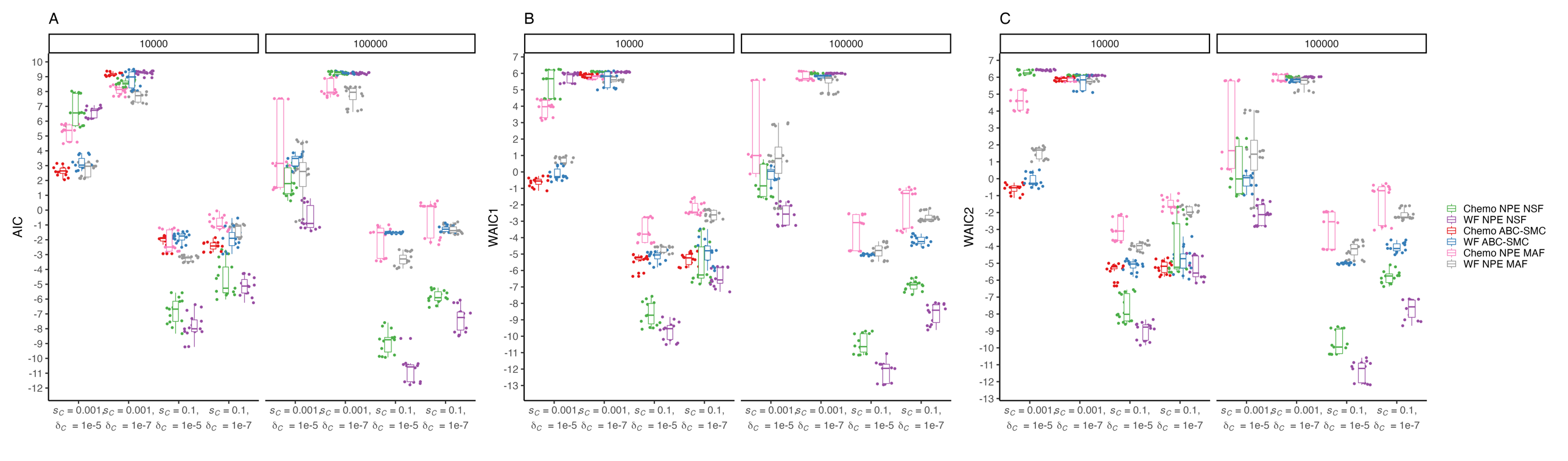

Supplement: S4 Fig — WAIC and AIC (lower is better) of models fitted on single synthetic observations using either the WF or chemostat (Chemo) model and either ABC-SMC or NPE for different combinations of fitness effect sC and formation rate δC with simulation budgets of 10,000 or 100,000 simulations per inference procedure (facets). We were unable to complete ABC-SMC with the chemostat model (red) when the training budget was 100,000 within a reasonable time frame. Data and code required to generate this figure can be found at https://doi.org/10.17605/OSF.IO/E9D5X. ABC-SMC, Approximate Bayesian Computation with Sequential Monte Carlo; AIC, Akaike information criterion; NPE, Neural Posterior Estimation; WAIC, widely applicable information criterion; WF, Wright–Fisher. (PNG) [file pbio.3001633.s006.png]

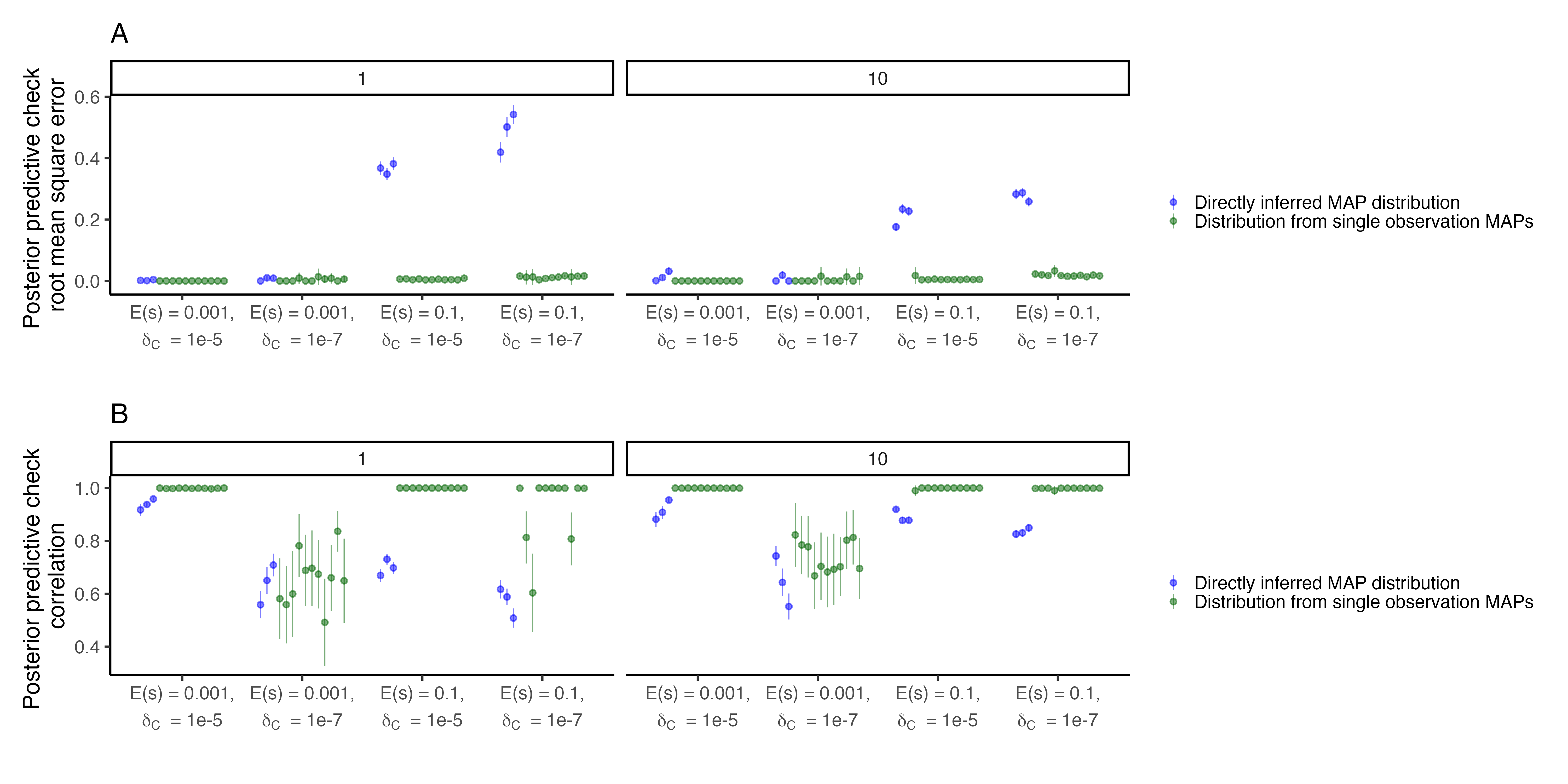

Supplement: S7 Fig — The x-axis shows the number of replicate simulated synthetic observations for a combination of parameters, and the y-axis shows the cumulative number of simulations needed to infer posteriors for an increasing number of observations (see the “Overview of inference strategies” section for more details), for observations with different combinations of CNV selection coefficient sC and CNV formation rate δC (A–D). Each facet represents a total simulation budget for NPE, or the maximum number of accepted simulations for ABC-SMC. Since NPE uses amortization, a single amortized network is trained with 10,000 or 100,000 simulations, and that network is then used to infer posteriors for each observation (note that a single amortized network was used to infer posteriors for all parameter combinations.) For ABC-SMC, each observation requires a separate inference procedure to be performed individually, and not all generated simulations are accepted for posterior estimation; therefore, the number of simulations used for a single observation may be more than the acceptance threshold, and the number of simulations needed increases with the number of observations for which a posterior is inferred. Data and code required to generate this figure can be found at https://doi.org/10.17605/OSF.IO/E9D5X. ABC-SMC, Approximate Bayesian Computation with Sequential Monte Carlo; CNV, copy number variant; NPE, Neural Posterior Estimation. (PNG) [file pbio.3001633.s009.png]

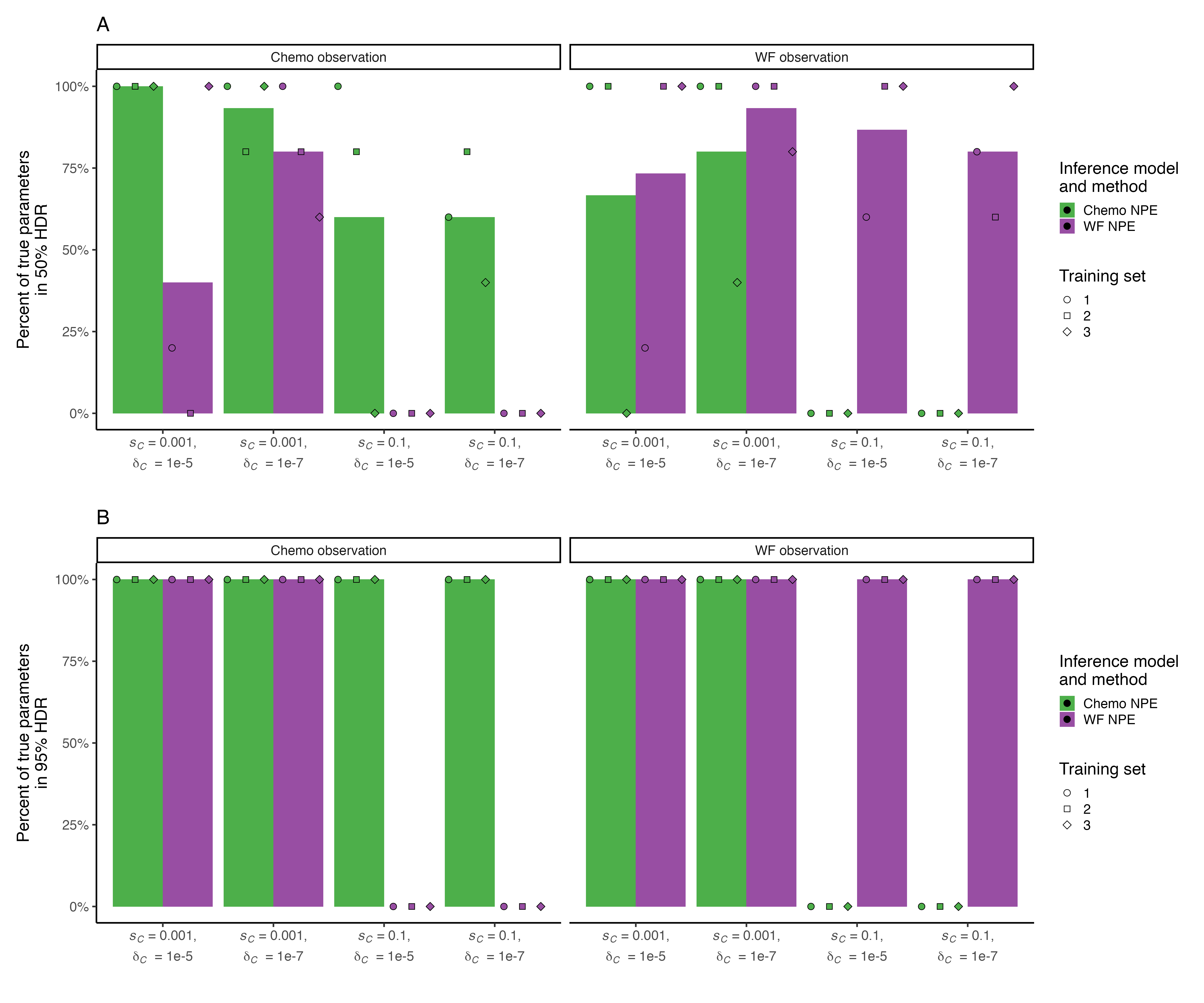

Supplement: S8 Fig — We performed inference on each synthetic observation using both models. For NPE, each training set corresponds to an independent amortized posterior trained with 100,000 simulations, with which each synthetic observation was evaluated. (A) Percentage of true parameters within the 50% HDR. The bar height shows the average of 3 training sets. (B) Percentage of true parameters within the 95% HDR. The bar height shows the average of 3 training sets. Data and code required to generate this figure can be found at https://doi.org/10.17605/OSF.IO/E9D5X. HDR, highest density region; NPE, Neural Posterior Estimation; WF, Wright–Fisher. (PNG) [file pbio.3001633.s010.png]

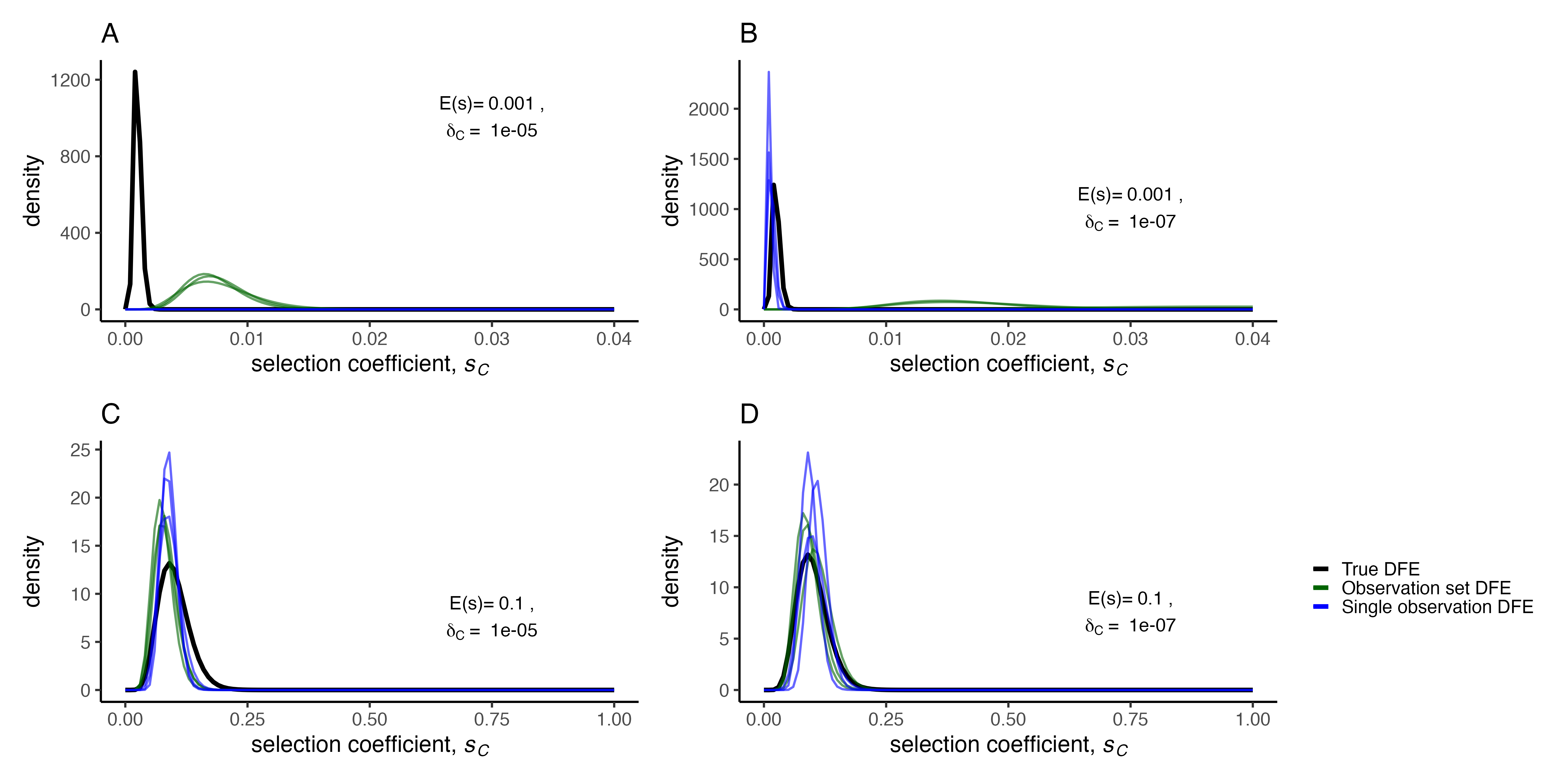

Supplement: S9 Fig — The MAP DFEs (blue curves) were directly inferred using 3 different subsets of 8 out of 11 synthetic observations. We also inferred the selection coefficient for each observation in the set of 11 individually, and fit Gamma distributions to sets of 8 inferred selection coefficients (green curves). All inferences were performed with NPE using the same amortized network to infer a posterior for each set of 8 synthetic observations or each single observation. Data and code required to generate this figure can be found at https://doi.org/10.17605/OSF.IO/E9D5X. DFE, distribution of fitness effects; MAP, maximum a posteriori; NPE, Neural Posterior Estimation; WF, Wright–Fisher. (PNG) [file pbio.3001633.s011.png]

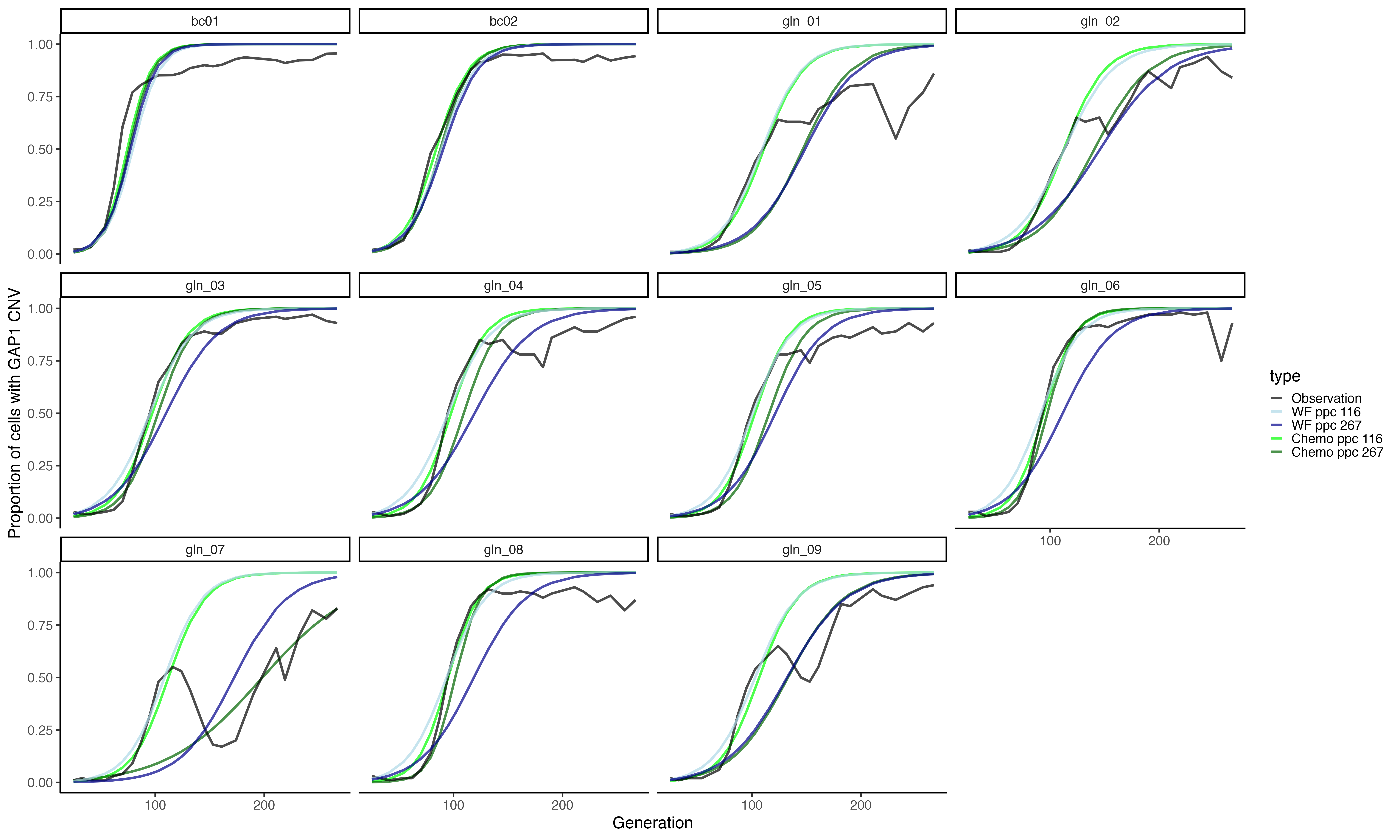

Supplement: S11 Fig — Proportion of the population with a GAP1 CNV in the experimental observations (black) and in posterior predictions using the MAP estimate shown in panels A and B with either the WF or chemostat (Chemo) model. Inference was performed with all data up to generation 267 (WF ppc 267, Chemo ppc 267), or excluding data after generation 116 (WF ppc 116, Chemo ppc 116). Formation rate and fitness effect of other beneficial mutations set to 10−5 and 10−3, respectively. Data and code required to generate this figure can be found at https://doi.org/10.17605/OSF.IO/E9D5X. MAP, maximum a posteriori; WF, Wright–Fisher. (PNG) [file pbio.3001633.s013.png]

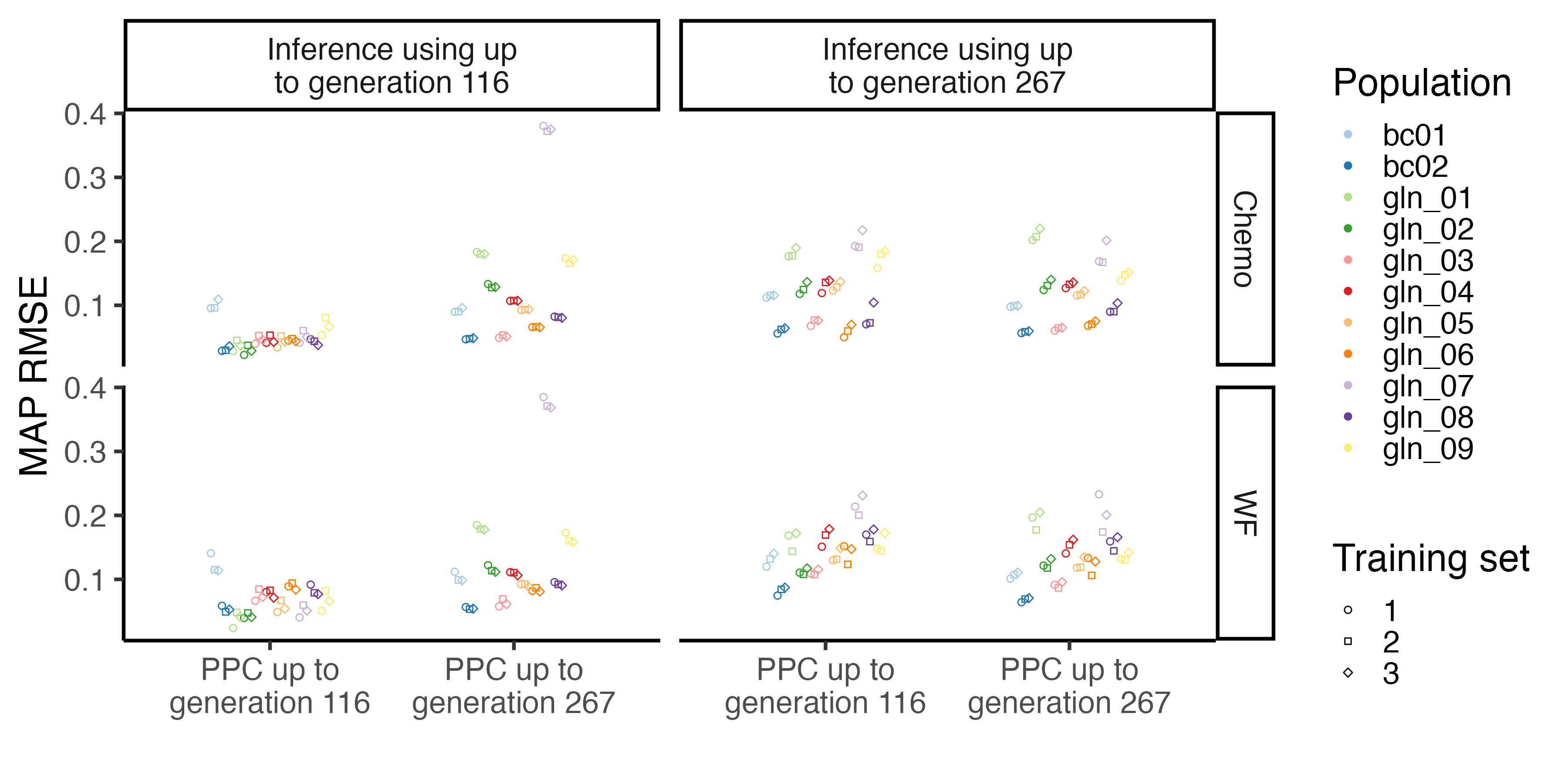

Supplement: S12 Fig — MAP posterior prediction RMSE when inference was performed excluding data after generation 116 (left) or using all data up to generation 267 (right). RMSE was calculated using either the first 116 generations or using up to generation 267 (x-axis). Data and code required to generate this figure can be found at https://doi.org/10.17605/OSF.IO/E9D5X. MAP, maximum a posteriori; RMSE, root mean square error. (PNG) [file pbio.3001633.s014.png]

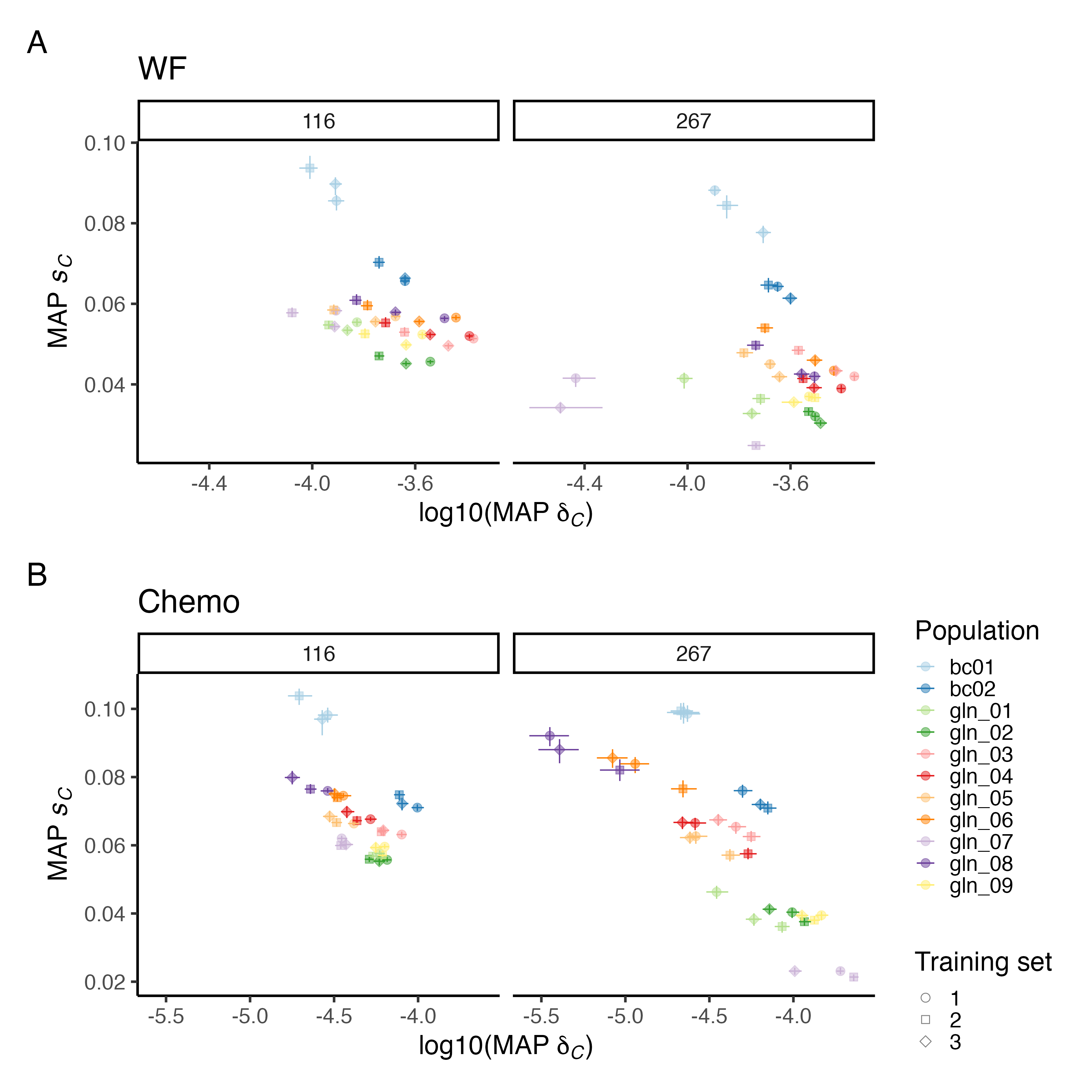

Supplement: S13 Fig — The inferred MAP estimate and 95% HDIs for fitness effect sC and formation rate δC, using the (A) WF or (B) chemostat (Chemo) model and NPE for each experimental population from Lauer and colleagues (2018). Inference was either performed with data up to generation 116 or with all data, up to generation 267 (facets). Each training set corresponds to 3 independent amortized posterior distributions estimated with 100,000 simulations. Data and code required to generate this figure can be found at https://doi.org/10.17605/OSF.IO/E9D5X. HDI, highest density interval; MAP, maximum a posteriori; NPE, Neural Posterior Estimation; WF, Wright–Fisher. (PNG) [file pbio.3001633.s015.png]

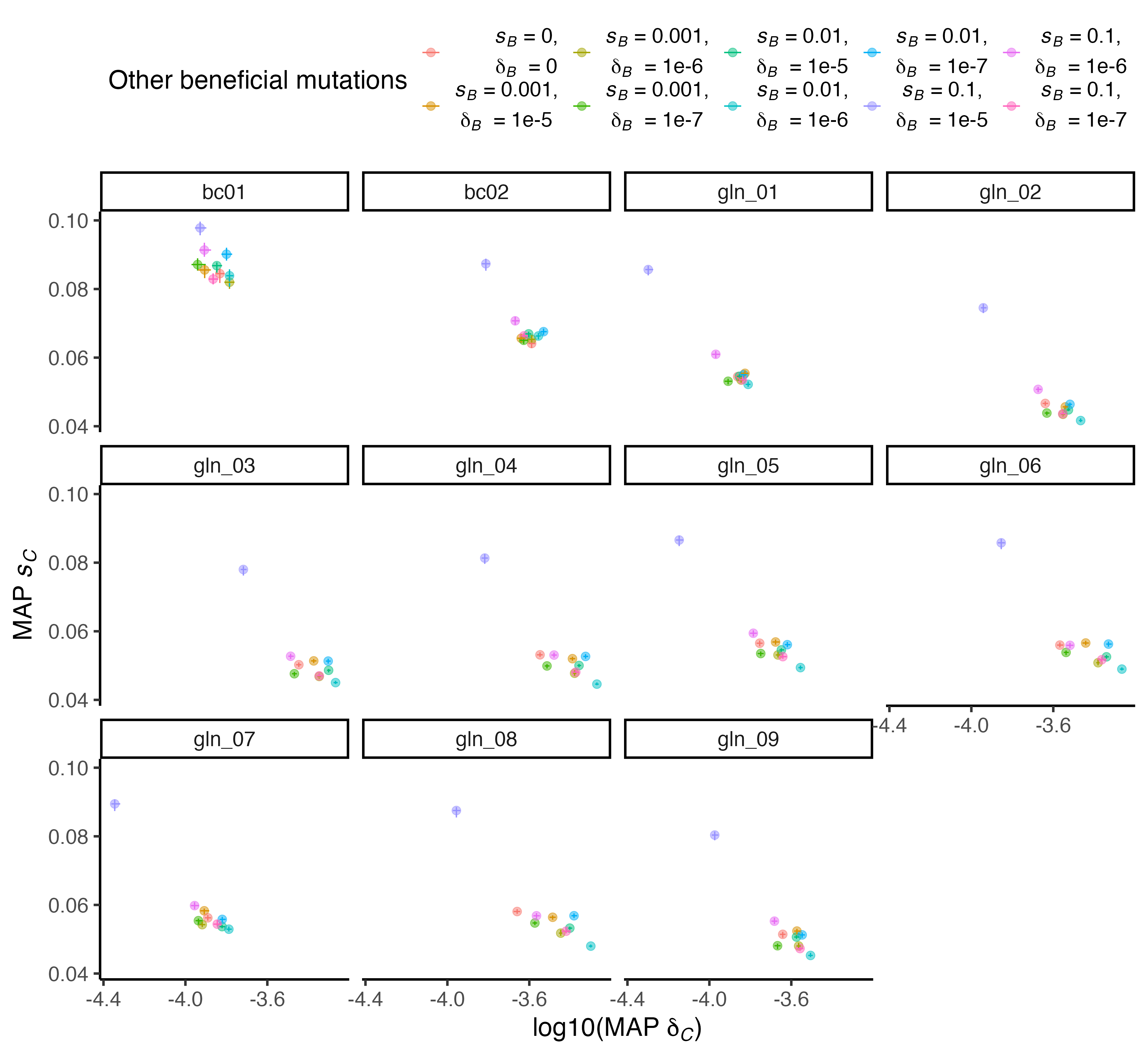

Supplement: S14 Fig — GAP1 CNV formation rate and selection coefficient inferred using NPE with the WF model does not change considerably when other beneficial mutations have different selection coefficients sB and formation rates δB, except when both sB and δB are high (purple). Data and code required to generate this figure can be found at https://doi.org/10.17605/OSF.IO/E9D5X. CNV, copy number variant; NPE, Neural Posterior Estimation; WF, Wright–Fisher. (PNG) [file pbio.3001633.s016.png]

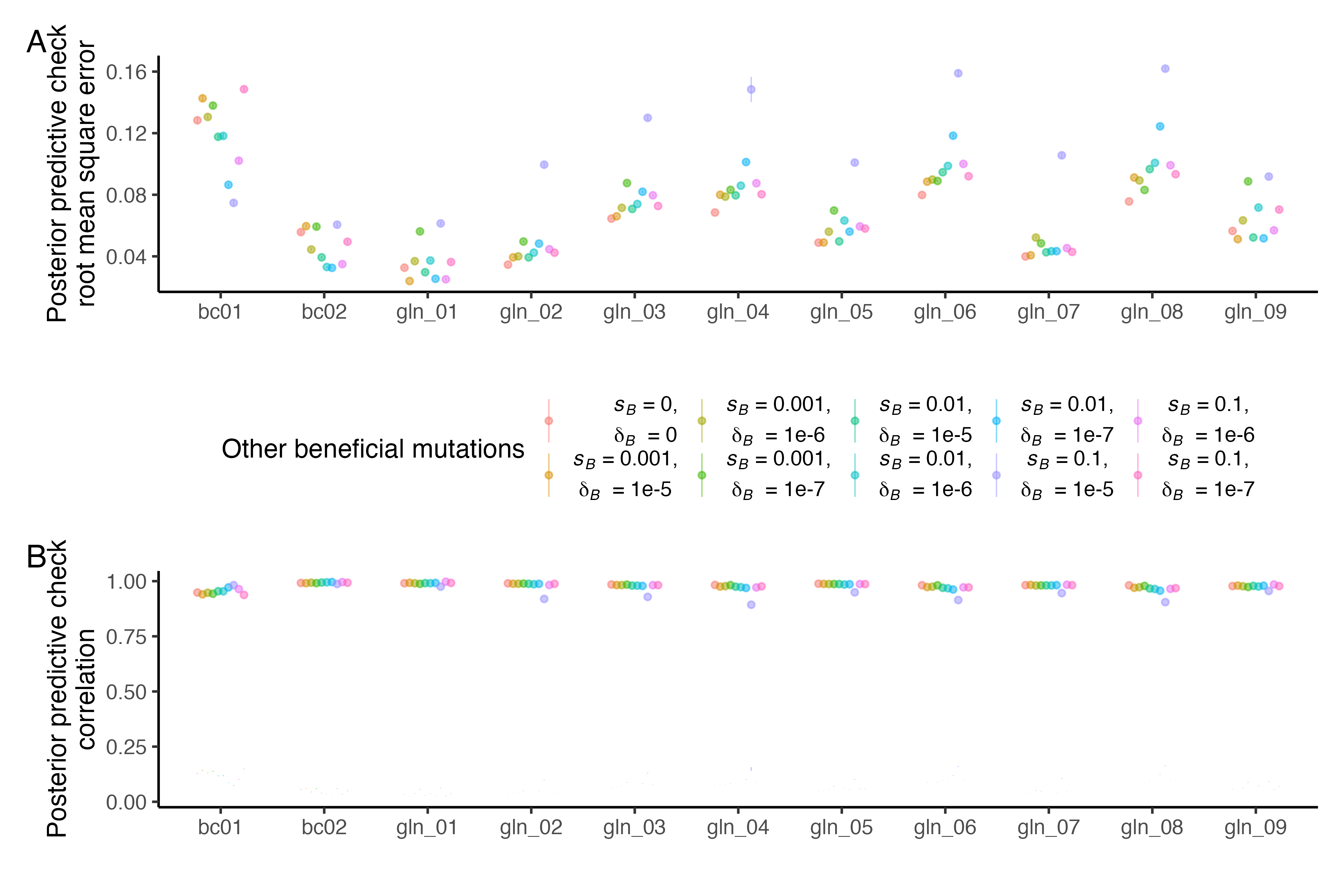

Supplement: S15 Fig — Mean and 95% confidence interval for RMSE (A) and correlation (B) of 50 posterior predictions compared to empirical observations up to generation 116, using posterior distributions inferred when other beneficial mutations have different selection coefficients sB and formation rates δB. Data and code required to generate this figure can be found at https://doi.org/10.17605/OSF.IO/E9D5X. RMSE, root mean square error. (PNG) [file pbio.3001633.s017.png]
