# Supplementary material for: Neural networks enable efficient and accurate simulation-based inference of evolutionary parameters from adaptation dynamics
Source: PLoS Biol. 2022 May 27;20(5):e3001633. doi: 10.1371/journal.pbio.3001633 (PMC9140244; doi:10.1371/journal.pbio.3001633)
Supplement: Attachment — Submitted filename: Response to reviewers 2.pdf [file pbio.3001633.s020.pdf]

REVIEWER'S COMMENTS:

Reviewer #1:

I thank the authors for their clarifications. I am happy that NPE performs better for the specific task in hand than does an ABC approach.

Thank you. We are pleased to hear that.

To clarify my thoughts on the simplicity or otherwise of the problem, I agree with what I think de Sousa et al are saying, that the general problem of inferring the distribution of adaptive mutations available to natural selection is a difficult task. In addressing this question, their work, alongside that of Hegreness et al., and Barrick et al., both provides some insight while having limitations. For example, Barrick et al use a model which aims to identify only the first mutation to arise within each population; this throws away part of the data, and is almost certainly biased towards identifying mutations of stronger effect.

We agree with this statement.

What I am less convinced is a difficult task is the specific question of how to fit a two-parameter Wright-Fisher model to data describing the evolution of a system under the influence of a single adaptive variant. If the function being optimised is not too rugged, a variety of approaches should give a decent answer to this problem. I remain unconvinced that likelihood approaches are as bad as the reviewers suggest. For example, if the log of the likelihood were calculated, I would not expect zero likelihoods would be a huge problem. Likelihood-based methods do not prevent the calculation of measure of confidence; a likelihood function plotted over parameter space would look similar to the posterior distributions of Figure 2. Further, identifying an approximate likelihood model might not be an especially intractable problem; the likelihood of a copy number variant having a specific frequency within the population given an observation might not be strictly analytical, but seems far from impossible to model if we accept that any modelling process involves a degree of approximation.

We do not claim that likelihood approaches are bad *per se*. However, our study presents a method that circumvents the need to define a likelihood function. The method we present is a more flexible approach and, as such, we argue likely to be more generally applicable.

The matter of the simplicity of the problem is a somewhat tangential critique of the work described in this manuscript. The revised manuscript is an improvement on the previous one, and I am happy that for this specific question the NPE algorithm provides a useful insight into the specific benefit of CNV formation in the GAP1 locus of yeast. However I am unclear about whether the specific problem chosen is the best one with which to demonstrate the potential of NPE to solve more difficult problems within evolutionary inference. I would want to see applications of NPE to problems with larger numbers of parameters involved before accepting more general claims of its value for evolutionary inference.

We agree that it will be exciting to see NPE implemented to problems with larger numbers of parameters. Indeed, we plan to do this in future work. Our study lays the foundation for this challenge and will guide our own future research and that of others when choosing approaches to simulation based inference.

Minor comments:

I note the WF-ABC method (Foll 2015) as another example of using ABC methods to process data from evolutionary experiments.

Thank you for pointing us to this method. We have added a reference to the paper in our text.

COMMENTS FROM THE ACADEMIC EDITOR:
[identifies himself as Arjan de Visser]

In addition to the comments of reviewer #1, I would like to point the authors to recent work involving myself, where we used Wright-Fisher simulations together with machine learning methods to infer mutation rates and fitness effects of 3 major mutation classes (SNPs, indels and structural variants), that best explained the observed mean and variance of each the numbers of mutations of each type in 96 evolved genotypes (Schenk, Zwart et al. 2022 Nat Ecol Evol, early online). Our approach is an alternative to using marker trajectories, which they (and de Sousa et al., Hegreness et al. and Barrick et al.) use, which the authors may consider mentioning in the introduction.

Thank you for drawing our attention to this interesting study. We have now referenced it in our introduction.
