# Supplementary material for: Neural networks enable efficient and accurate simulation-based inference of evolutionary parameters from adaptation dynamics
Source: PLoS Biol. 2022 May 27;20(5):e3001633. doi: 10.1371/journal.pbio.3001633 (PMC9140244; doi:10.1371/journal.pbio.3001633)
Supplement: Attachment — Submitted filename: Response to reviewers.docx [file pbio.3001633.s019.docx]

Reviewer comments in black roman, *our response in blue italics*

Reviewer #1:

The following review comments are based upon version R1 of the manuscript.

Avecilla et al compare the results of two methods for evolutionary inference, as applied to learning the rate of generation, and the selective advantage, of copy number variants in a population. A method of neural posterior estimation (NPE) outperforms a method of Approximate Bayesian Computation (ABC). I have preliminary comments about the question addressed in this study, followed by comments about the manuscript itself.

Preliminary comments

P1: As I understand it, evolutionary inference involves fitting a model to data so as to estimate evolutionary parameters. This requires a choice of model e.g. Wright-Fisher, a distance function, which describes the closeness of the output of the model to the observed data, and a means via which the parameter space of the model can be explored.

*Whereas this is the commonly used approach (ABC), our study includes a method that does not require a distance function or means for exploring the parameter space. Instead, our approach directly approximates the joint distribution P(θ,X’) of simulator input θ (model parameters) and output X (data), and then conditions this distribution on the real data X to get the posterior distribution P(θ|X). Our study demonstrates that this method is more accurate and more efficient than the current state-of-the-art method used in the field.*

P2: As I understand it, the difference between the ABC and NPE methods that are applied here is the manner in which they explore parameter space. The models used and the distance functions are the same in each case.

*That is incorrect. NPE does not use a distance function. Moreover, it directly approximates the posterior density function, whereas ABC samples from the posterior; these samples are then used to estimate the posterior using methods such as kernel density estimation. Moreover, NPE does not require a proposal distribution. Both inference methods do use the same models.*

P3: The example problem given of inferring mutation rates and selection coefficients is relatively simple. The output from the model, describing the proportion of cells with GAP1 CNV, is one-dimensional, if time-dependent, while the space of inferred parameters is only two-dimensional, with a selection coefficient s_C, and a mutation rate \delta_C.

*We disagree with this statement. The problem is not simple and we note that Reviewer #3 agrees it is not simple. Though the output is one-dimensional, it is time-dependent and very stochastic as shown in Fig 1A. The parameter space is 2D, but the likelihood is intractable and the parameters are not completely identifiable.*

P4: Given point P3, I would expect a very simple model to produce a decent solution to this problem. For example, taking each replicate in turn, I expect that it would be possible to propose initial values of {s_C, \delta_C}, then iteratively change these values in the manner of a downhill optimisation (i.e. accepting new parameters that give smaller distances between the model and the data) so as to infer optimal parameters for each replicate.

*We assume that the reviewer means “simple method” rather than “simple model”. We strongly disagree with this expectation for four reasons. First, the suggested approach (downhill optimization / gradient descent) would not provide a measure of confidence for the inferred “optimal” parameters, making the resulting point estimate difficult to interpret. Second, the proposed approach would be highly computationally intensive, requiring a vast number of simulations to converge, as most parameter choices would yield zero likelihood. To show that this is indeed the case we have now performed rejection ABC, which is the simplest ABC method, and compared the posterior estimate to the posteriors inferred with NPE (below), and find that NPE performs as well or better than rejection ABC. We do not think this additional comparison adds to the study, so we have not included it in the main text, but are happy to do so if requested. Third, the likelihood is intractable for the proposed approach, so a distance function must be used, which makes the process biased if the distance is not well chosen. Finally, it is well known that the problem of joint inference of evolutionary parameters is not simple, and prior studies have pointed out the difficulty of solving this problem, for example:*

- *Moura de Sousa JA, Campos PRA, Gordo I (2013) An ABC method for estimating the rate and distribution of effects of beneficial mutations. Genome biology and evolution, 5(5):794–806.*
- *Hegreness M, Shoresh N, Hartl D, Kishony R (2006) An equivalence principle for the incorporation of favorable mutations in asexual populations. Science, 311(5767):1615–1617.*
- *Barrick, J. E., Kauth, M. R., Strelioff, C. C. & Lenski, R. E. Escherichia coli rpoB mutants have increased evolvability in proportion to their fitness defects. Mol. Biol. Evol. 27, 1338–1347 (2010).*

**NPE outperforms rejection ABC for twelve parameter combinations using the Wright-Fisher model.** Results of inference on five simulated synthetic observations using the Wright-Fisher (WF) model for different combinations of fitness effect *s_C_* and mutation rate 𝛿_C_. Simulations and inference were performed using the same model. For NPE, each training set corresponds to an independently amortized posterior distribution trained on a different set of 100,000 simulations, with each synthetic observation evaluated to produce a separate posterior distribution. For rejection ABC, each observation was compared to 100,000 simulations using an Euclidean distance function. The 5% of simulations that had the smallest distance were kept to approximate the posterior distribution. Each training set is a comparison to a different set of 100,000 simulations. **A)** The percent of true parameters within the 50% or 95% HDR of the inferred posterior distribution. The bar height shows the average of three training sets. Distribution of widths of 95% highest density interval (HDI) of the posterior distribution of the **B**) fitness effect *s_C_* and **C**) CNV mutation rate 𝛿_C_ (, calculated as the difference between the 97.5 percentile and 2.5 percentile, for each separately inferred posterior distribution. **D)** Log-ratio (relative error) of MAP estimate to true parameter for *s_C_* and 𝛿_C_. Note the different y-axis ranges. A perfectly accurate MAP estimate would have a log-ratio of zero. **E)** Mean and 95% confidence interval for correlation coefficient of 50 posterior predictions compared to the synthetic observation for which inference was performed. **F)** Correlation coefficient of posterior prediction posterior prediction generated with MAP parameters compared to the synthetic observation for which inference was performed.

**NPE outperforms rejection ABC for four parameter combinations using the chemostat model.** Results of inference on five simulated synthetic observations using the chemostat (Chemo) model for different combinations of fitness effect *s_C_* and mutation rate 𝛿_C_. Simulations and inference were performed using the same model. For NPE, each training set corresponds to an independently amortized posterior distribution trained on a different set of 100,000 simulations, with each synthetic observation evaluated to produce a separate posterior distribution. For rejection ABC, each observation was compared to 100,000 simulations using an Euclidean distance function. The 5% of simulations that had the smallest distance were kept to approximate the posterior distribution. Each training set is a comparison to a different set of 100,000 simulations. **A)** The percent of true parameters within the 50% or 95% HDR of the inferred posterior distribution. The bar height shows the average of three training sets. Distribution of widths of 95% highest density interval (HDI) of the posterior distribution of the **B**) fitness effect *s_C_* and **C**) CNV mutation rate 𝛿_C_ (, calculated as the difference between the 97.5 percentile and 2.5 percentile, for each separately inferred posterior distribution. **D)** Log-ratio (relative error) of MAP estimate to true parameter for *s_C_* and 𝛿_C_. Note the different y-axis ranges. A perfectly accurate MAP estimate would have a log-ratio of zero. **E)** Mean and 95% confidence interval for correlation coefficient of 50 posterior predictions compared to the synthetic observation for which inference was performed. **F)** Correlation coefficient of posterior prediction posterior prediction generated with MAP parameters compared to the synthetic observation for which inference was performed.

Comments:

1. While I appreciate the value of ABC methods in cases where a likelihood function is intractable I am not convinced that this is true for a Wright-Fisher model.

*The likelihood function of the WF model is intractable. Indeed, Reviewer #3 agrees and has published papers supporting this. In addition, we also tested the more complex chemostat model, and NPE was superior to ABC-SMC for inference using this model as well. As reviewer 2 points out, we show that Wright-Fisher can be used to approximate the chemostat in some, but not all cases, and our results indicate that Wright-Fisher is not the best-suited model to use in all real-world cases; specifically, if many beneficial CNVs turn out to have strong selection coefficients. We believe that we have shown the general applicability of NPE.*

Where allele frequency data is collected from a population using genome sequencing of representative samples from a population, the output would be expected to approximate a binomial sample under perfect sequencing and sampling, or an overdispersed binomial sample given errors. Where short reads are collected from a large part of the genome that is subject to copy number variation, the number of reads from that region would be expected to approximate a Poisson distribution under perfect sampling, or in reality more likely an overdispersed Poisson model. The variances of distributions can be estimated via repeat sampling or variance from deterministic behaviour;

*Our data are not short-read sequencing data, but use our CNV reporter system for quantifying CNV dynamics. Regardless, the issue is not the stochasticity of experimental sampling, rather , it is the stochasticity due to mutation and drift and non-linearity due to selection inherent in the model.*

under these circumstances, and given the desire to estimate a posterior distribution that reflects the extent of uncertainty in the data, is there an advantage to a likelihood-free approach?

*Because the likelihood is intractable, and cannot be directly computed, a likelihood-based approach is not feasible. Approximate likelihood approaches can be used, for example by estimating the likelihood using simulations, but they will be more computationally intensive than our likelihood-free approach. Simpler models, for example without selection, can apply analytic approximations (e.g. Tataru, Bataillon & Hobolth 2015), but selection, because it is non-linear, makes such approaches less appealing.*

2. The results section says that interpolation was used to match time points between experiments. I am concerned about the effect of this on the biological inferences made. When making estimates from these data (as opposed to testing on real data) could the methods be applied simply to the data?

*We have now clarified the impact of interpolation, which was used to create uniform datasets across our different experiments. The following plot and legend have been added to the supplementary material as Supplementary Figure 1 to show the interpolated data and how it differs from the originally collected data for bc01 and bc02. As can be seen in the plot, the interpolated values are in good agreement with the empirical values.*

***Supplementary figure 1.*** *Populations gln01-gln09 and bc01-bc02 have different timepoints - the gln populations have 25 timepoints in total, whereas the bc populations have 32 timepoints in total. Of these, 12 of the timepoints are the same in both populations. To match the timepoints in the gln populations, we interpolated from the two nearest timepoints in the bc populations (using pandas.DataFrame.interpolate(‘values’)). This way, we can use the same data (same timepoints) for inference for all 11 populations so that we can use the same amortized NPE posterior to infer parameters for both gln populations and bc populations. Original bc data is shown as black dots, the matched data, with interpolated timepoints, is shown as red crosses. Code used to perform interpolation can be found at* [*https://github.com/graceave/cnv_sims_inference/*](https://github.com/graceave/cnv_sims_inference/blob/master/inference/Interpolating_bc.ipynb)

3. I am concerned about what precisely can be learnt from the comparison of the two methods. While it is claimed that the NPE method outperforms the ABC method, and results are shown to demonstrate this, I don't understand why the ABC method does worse. Given the simplicity of the problem, what is going wrong?

*The reviewer is incorrect in assuming that the problem is simple. Our explanation for the difference in the performance is that the NPE approach is superior as it directly approximates the joint distribution of the simulator, rather than attempting to approximately sample from the posterior. This provides greater efficiency and accuracy as outlined in a recent review of simulation-based inference (Cranmer et al. 2020).*

Could it be that:

i) The ABC method is equally as good, but is computationally less efficient? For example, running the ABC method for more iterations gave a better result. Is ABC bad at exploring parameter space, or just slow to converge?

*It is certainly true that ABC is less efficient, and this is a major consideration for suggesting the use of NPE. To support this claim we have now added supplementary figure 7 visualizing the number of simulations needed for inference compared with the number of observations for which a posterior can be inferred. We have added the following statements to the main text:*

*In the section, “Overview of inference strategies”:*

*“The number of samples (i.e., simulations used for inference) drawn from the proposal distribution during each iteration of ABC-SMC was determined according to the number of samples needed to approximate the next posterior, which is adapted based on the shape of the current posterior distribution* [*[92]*](https://paperpile.com/c/paszxb/oaMZ) *with a maximum number of samples per iteration set based on the total simulation budget (see Methods). We applied bounds on the maximum number of samples used to approximate the posterior in each iteration; however, the total number of samples (simulations) used in each iteration is greater because not all simulations are accepted for posterior estimation (see Methods). For each observation, we performed ABC-SMC with multiple iterations until either the acceptance threshold (ε = 0.002) was reached or until 10 iterations had been completed. We performed inference on each observation independently three times. Though we refer to different observations belonging to the same “training set”, a different ABC-SMC procedure must be performed for each observation.”*

*In the section, “NPE outperforms ABC-SMC”:*

*“We performed NPE using 10,000 or 100,000 simulations to train the neural network and found that increasing the number of simulations did not substantially reduce the MAP estimation error, but did tend to decrease the width of the 95% HDIs for both parameters (Supplementary Figure 6). Similarly, we performed ABC-SMC with per observation maximum accepted budgets of 10,000 and 100,000 parameter samples (i.e. “particles” or “population size”) numbers of 10,000 and 100,000, which corresponds to increasing number of simulations per inference procedure, and found that increasing the budget decreased the estimation error and widths of the 95% HDIs for both parameters (Supplementary Figure 6). Overall, amortization with NPE allowed for more accurate inference using fewer simulations and less computation time (Supplementary Figure 7).”*

**Supplementary Figure 7. The cumulative number of simulations needed to estimate posterior distributions for multiple observations.** The x axis shows the number of replicate simulated synthetic observations for a combination of parameters and the y axis shows the cumulative number of simulations needed to infer posteriors for an increasing number of observations *(*see *Overview of inference strategies* for more details), for observations with different combinations of CNV selection coefficient s_C_ and CNV formation rate 𝛿_C_ (**A-D**). Each facet represents a total simulation budget for NPE, or the maximum number of accepted simulations for ABC-SMC. Since NPE uses amortization, a single amortized network is trained with 10,000 or 100,000 simulations, and that network is then used to infer posteriors for each observation (note that a single amortized network was used to infer posteriors for all parameter combinations.) For ABC-SMC, each observation requires a separate inference procedure to be performed individually, and not all generated simulations are accepted for posterior estimation; therefore, the number of simulations used for a single observation may be more than the acceptance threshold, and the number of simulations needed increases with the number of observations for which a posterior is inferred.

ii) Both methods are equally good, but the specific implementation of the ABC method you are using is not a good one, for example in terms of not having been coded very well or in an efficient manner?

*ABC-SMC is a very well-established method (Sisson et al 2007). We use pyABC, which is a stable framework for performing ABC-SMC, widely used in a variety of applications (see* [*https://pyabc.readthedocs.io/en/latest/references.html*](https://pyabc.readthedocs.io/en/latest/references.html)*).*

While I accept the results of the comparison, I am left unconvinced as to whether NPE is particularly good, or whether ABC is just extraordinarily bad. Further, while I am aware of the potential for neural networks in machine learning applications I am also unconvinced about whether this particular application is a case of using an overly-complicated technology to solve a very simple problem. Is there a reason why naive parameter optimisation would not work for these data?

*We do think there are good reasons why naive parameter optimization does not work. Namely, the generative model is not simple, the data are stochastic, and the NPE method is not overly-complicated but rather a natural, though technically advanced, extension of previous likelihood-free inference methods that used regression-based approaches (e.g. Beaumont, Zhang, Balding 2002). To avoid similar conclusions from the readers, we have substantially revised the introduction to the paper to give a better overview so that readers can appreciate the difficulty of estimating evolutionary parameters from these data.*

Minor comments:

1. Where details are given about numbers of iterations and so forth, it would be valuable to have a measure of the actual time taken to run the optimisation i.e. how many minutes and on what sort of machine.

*The majority of running time is for simulations. Therefore, we have added Supplementary Table 1 which reports how long a single simulation takes using each model (Wright-Fisher or chemostat) for 4 combinations of parameters.*

**Supplementary Table 1. Wall time to run one simulation.** Running time for a single Wright-Fisher simulation or a single chemostat simulation for each of the following parameter combinations on a 2019 MacBook Pro operating Mac OS Catalina 10.15.7 with a 2.6 GHz 6-Core Intel Core i7 processor. Code used to determine these times can be found at <https://github.com/graceave/cnv_sims_inference/blob/master/inference/Simulation_time.ipynb>

| **Model** | **𝛿_C_** | **s_C_** | **Wall time (seconds)** |
| --- | --- | --- | --- |
| Wright-Fisher | 0.1 | 10^-5^ | 0.0136 |
| Chemostat | 0.1 | 10^-5^ | 10.8608 |
| Wright-Fisher | 0.1 | 10^-7^ | 0.0099 |
| Chemostat | 0.1 | 10^-7^ | 11.2390 |
| Wright-Fisher | 0.001 | 10^-5^ | 0.0108 |
| Chemostat | 0.001 | 10^-5^ | 11.3547 |
| Wright-Fisher | 0.001 | 10^-7^ | 0.0086 |
| Chemostat | 0.001 | 10^-7^ | 10.6964 |

2. The abstract mentions yeast. Is this S. cerevisiae?

*Yes, “yeast” is the commonly used name for S. cerevisiae, we have changed this in the abstract.*

Reviewer #2:

This manuscript presents a thorough analysis of the use of likelihood-free methods to infer the formation rate and fitness effects of CNVs from adaptive evolution experiments in which a fluorescent reporter is used to quantify CNV dynamics. The authors show based on simulations that CNV formation rates and fitness effects can be determined accurately using likelihood-free inference on both Wright-Fisher and chemostat models, except if the CNV has a very low formation rate and selection coefficient. Neural posterior estimation (NPE) was found to outperform approximate Bayesian computation with sequential Monte Carlo (ABC-SMC) as a likelihood-free inference algorithm. Furthermore, the authors validate their approach on experimental data, showing that NPE inference under Wright-Fisher or chemostat models yields similar selection coefficient estimates as obtained through barcode-based lineage tracking and pairwise competition assays.

Overall, the study convincingly shows that NPE-based likelihood-free inference is well-suited to obtain CNV formation rates and fitness effects from chemostat adaptive evolution experiments using fluorescent reporters. These results also open perspectives for efficient determination of mutation rates and selection coefficients in other experimental evolution setups. In brief, the methodology outlined here may be very valuable to the experimental evolution community.

*We thank the reviewer for the favorable assessment of our manuscript and recognizing its broader value to the experimental evolution community. We are grateful for the constructive comments of the reviewer.*

The setup of the study is well thought-out and the manuscript is well-written. I have no major comments on the analyses performed or their presentation, but I do have a couple of minor points I feel should be addressed:

- While performing inference on a set of observations, the authors conclude that estimating the DFE through inference of individual selection coefficients from each observation is superior to inference of the distribution from multiple observations. Any suggestions on why this might be ?

*We agree that this is not necessarily intuitive and our conclusion is based on empirical evidence. We do not have a comprehensive explanation for this observation, although it is likely because the DFE estimation from multiple observations might bias towards the strong-effect mutations.*

-While inferring the GAP1 CNV selection coefficient and formation rate from empirical data, the authors find that the inferred GAP1 CNV formation rate under the Wright-Fisher model is higher than under the chemostat model. It might be worth pointing out that this is consistent with the overprediction of formation rates under Wright-Fisher in the simulation results when selection coefficients are high. This does suggest that Wright-Fisher is not the best-suited model to use in all real-world cases, in particular if many beneficial CNVs would turn out to have strong selection coefficients. Maybe this should be mentioned more explicitly.

*Thank you for this point. We have now addressed this in the discussion:*

*“Although a more complex evolutionary model better describes the evolution experiments performed in chemostats, we find that a standard Wright-Fisher model can be a sufficient approximation for inference using NPE. However, the inferred GAP1 CNV formation rate under the Wright-Fisher model is higher than under the chemostat model (Figure 6A-B), which is consistent with the overprediction of formation rates using the Wright-Fisher model for inference when an observation is generated by the chemostat model and selection coefficients are high (Figure 4C-D). This suggests that Wright-Fisher is not the best-suited model to use in all real-world cases, in particular if many beneficial CNVs turn out to have strong selection coefficients.”*

-Why was only bc01 used for lineage tracking and not bc02 ?

*This exclusion of bc02 was due to experimental issues. We attempted to analyze both populations for lineage tracking. However, when analyzing bc02 we obtained low DNA yields from frozen population samples resulting in very low sequencing coverage that was not amenable to DFE inference. Because of the concordance between lineage tracking and competitive fitness assays we do not think the inclusion of the bc02 DFE strengthens the findings.*

-	Figure 6 : add labels for training sets

-	'The difference between any combination of model and method was less than 2 for δC=10-5 and sC=0.001' : 10-5 needs to be 10-7.

-	reference to 'Figure 2C, Supplementary XX' and several references to 'Supplementary Files' need to be amended

-	labels of Supp Fig 3 partially fall off page.

-	'...as well or better than direct inference of the DFE from a set of observations for both α = 1 (an exponential distribution) and α = 10 (sum of ten exponentials) (Figure 5)' : refer to Supp Fig 6 here as well.

*We have fixed the above errors, thank you!*

-	Supp Fig 13 : I don't understand what μ(s) is exactly, how it was inferred from the data, and why this plot is a histogram rather than a dot plot.

*We agree with the reviewer that this plot is confusing, and as we do not think it is necessary for readers to understand the paper, we have removed it.*

-	The formulas for the frequencies of different genotypes after mutation in the Evolutionary models section of the Methods contains some errors. δN in the first equation should be δB, xC in the second equation should be xB and xN in the third equation should be xC.

*We have fixed these errors, thank you!*

-	It is unclear where the formula for estimating the effective population size in the chemostat comes from. There seems to be a factor 2 missing in front of var(p'|p) and the averaging likely applies to the entire fraction rather than only the denominator. Additionally, the harmonic mean may be preferable for averaging over generations rather than the arithmetic mean.

*We have added a citation to J. F. Crow, M. Kimura, An introduction to population genetics theory (1970) for this formula.*

*In Crow and Kimura, pg 327, eq 7.3.2 is: Vx = p(1-p)/2Ne. The factor of 2 is for diploids, but as we are considering haploids, we remove this factor. Thus, Ne = p(1-p)/Vx.*

*To estimate Vx, we take the average over all generations. We verified this approach: simulating WF model, we estimate Ne equal to the population size parameter of the model (this verification can be found in the notebook used to perform this estimation, which can be found at https://github.com/graceave/cnv_sims_inference/). The harmonic mean is usually used to estimate effective population size in a population with variable population sizes by taking the harmonic mean of the population sizes. However, the mean we take here is to estimate the variance in frequency of a minor allele in a population of (almost) constant population size (in a chemostat). So we do not think the harmonic mean is needed.*

-	'If a clone was barcoded, relative fitness using linear regression of the natural logarithm of the ratio of the barcoded GAP1 CNV containing clone to the unlabeled ancestor, and the natural logarithm of the ratio of the unevolved barcoded GAP1 CNV reporter ancestor to the unlabeled ancestor against the number of elapsed hours, adding an additional interaction term for the evolved versus ancestral state.' : something is missing in this sentence.

*Thank you. We have corrected this sentence to now read: “If a clone was barcoded, relative fitness was computed using linear regression of the log-ratio of the frequencies of the barcoded GAP1-CNV-containing clone and the unlabeled ancestor, and the log-ratio of the frequencies of the unevolved barcoded GAP1 CNV reporter ancestor to the unlabeled ancestor against the number of elapsed hours, adding an additional interaction term for the evolved versus ancestral state.”*

-	'For each lineage in the barcoded population, beneficial mutations continually occur at a total beneficial mutation rate Ub, with fitness effect s, with a certain spectrum of fitness effects of mutations —(s).' : something is off in this sentence.

*We have amended the sentence to read: “For each lineage in the barcoded population, beneficial mutations continually occur at a total beneficial mutation rate Ub, with fitness effect s, which results in a spectrum of fitness effects of mutations 𝜇(s).”*

Reviewer #3:

[see also the attached marked up version of the manuscript PDF]

General comments

The submitted ms focuses on a specific question within the field of the genetics of adaptation in (completely asexual ) populations: estimating the mutation rate and selective effect of mutation at a specific locus while treating the rest of the genome "separately". This is warranted especially if one knows in advance that most of the action can be traced back to the locus of interest. The methods developed here are heavily inspired by a specific "yeast settings" that looks promising to track evolution in real time but where it is difficult to see how the system can give broad insights and how the methods developed and tested here can generalize to other settings.

*We thank the reviewer for noting the promise of our approach. The reviewer is correct in stating that our research is specific to asexual reproduction. However, we strongly disagree that the method is not relevant to other settings. Asexual reproduction is the dominant mode of reproduction for almost all microbes and relevant to diverse scenarios from tumor evolution to viral evolution. We expect that our research will have a broad impact on efforts in these diverse contexts.*

Major issues.

A. The element that I find most limiting currently is that the inference methods that are presented here only are estimating two parameters: the rate of mutation at a specific locus (here the gap1 CNV) and effect of a beneficial mutation at this locus. All inference is predicated on the assumption that all other important parameters are "known" independently . This includes at least three critical evolutionary parameters that are notoriously difficult to estimate from data:

- the genome wide mutation rates at other loci in the genome

- the distribution of fitness effects of these "other" mutations

- the effective size realized during the whole experiment.

This makes for a very idiosyncratic study where it is hard generalize / gain insights for to a broader range of situations in experimental evolution.

*We disagree that joint estimation of fitness effect and mutation rate at a single locus is idiosyncratic. Indeed, there are multiple papers that have previously attempted to do this, making our study of broad interest. The reviewer is correct that our study has focused on estimation of two parameters whereas additional evolutionary parameters contribute to the overall evolutionary dynamics. We plan to extend the analysis to additional parameters estimation problems in future work based on the foundational work reported in this manuscript.*

B. The methodology used for comparing inference methods (ABC versus NPE) and to appreciate how the different estimators are behaving is very difficult to follow ( scattered between an overview , a method and numerous figures and supplementary figure legends),

*We have revised the text throughout to make the methodology easier to follow.*

but after reading through carefully a few points are really baffling me :

In short :

1. it is hard to see how the two methods ( SMS-ABC and NPE) are compared on equal foots (in term of amount of computation made available to ensure that methods return sensible approximate posterior distribution) (see numerous comments to the authors in the annotated PDF) . It seems that ABC-SMC typically needs at least 10^5 simulations to approximate sensibly posteriors but it seems that in the main figure ( figure3) only a tenth are allowed (if I understood the figure legend) .

*The reason that we showed 10^4 simulations is that running 10^5 simulations is computationally very expensive. Thus, a major advantage of NPE is that it requires considerably less computation time, especially when using amortization on replicates of the same experiment. Nevertheless, we have made a comparison for ABC-SMC with the Wright-Fisher model in Supplementary Figure 6 (previously supplementary figure 4, pasted below).Increasing the number of simulations does decrease the 95% HDI, but only decreases the MAP relative error when the selection coefficient is 0.1.*

***Supplementary Figure 4. Effect of simulation budget on relative error of MAP estimate and width of HDIs*** *for inference on single synthetic observations. For NPE, amortized posteriors were estimated using either 10,000 or 100,000 simulations, with which each synthetic observation was evaluated to produce a separate posterior distribution. For ABC-SMC, a posterior was independently inferred for each observation with a maximum of 10,000 or 100,000 total simulations accepted and a stopping criteria of 10 iterations or ε <= 0.002, whichever occurs first. The grey lines in (****A, D****) indicate a relative error of zero (i.e., no difference between MAP parameters and true parameters). (****D, E, F****) We were unable to complete ABC-SMC with the chemostat model (red) when the training budget was 100,000 within a reasonable time frame.*

2. It seems that the comparisons made are based on at best a handful (usually 5 max in some instance apparently 15 ) of truly independent datasets simulated on very specific scenarios. (see my numerous comments on Figure 3 and several suppl figures comparing the methods)

I think a disproportionate emphasis is put on claiming superiority of NPE relative to BAC-SMC . I really have no share on either method . I am open to the fact that NPE is potentially superior to ABC-SMC.. but I currently remain unconvinced (because of points 1 and 2 above).

*We have extended our analysis to additional cases using the Wright-Fisher model, and include those results in Supplementary Figure 5 (below) and Supplementary Figure 6 (above). These additional analyses provide further support for our claim that NPE is superior across a broad range of parameter values.*

***Supplementary Figure 5. NPE performs similar to or better than ABC-SMC for eight additional parameter combinations.*** *The figure shows the results of inference on five simulated synthetic observations using the Wright-Fisher (WF) model per combination of fitness effect s_C_ and mutation rate 𝛿_C_. Simulations and inference were performed using the same model. For NPE, each training set corresponds to an independently amortized posterior distribution trained on a different set of 100,000 simulations, with which each synthetic observation was evaluated to produce a separate posterior distribution. For ABC-SMC, each training set corresponds to independent inference procedures on each observation with a maximum of 100,000 total simulations accepted for each inference procedure and a stopping criteria of 10 iterations or ε <= 0.002, whichever occurs first.* ***A)*** *The percent of true parameters within the 50% or 95% HDR of the inferred posterior distribution. The bar height shows the average of three training sets.* ***B-C)*** *Distribution of widths of 95% highest density interval (HDI) of the posterior distribution of the fitness effect s_C_ (****B****) and CNV mutation rate 𝛿_C_ (****C****), calculated as the difference between the 97.5 percentile and 2.5 percentile, for each separately inferred posterior distribution.* ***D)*** *Log-ratio (relative error) of MAP estimate to true parameter for s_C_ and 𝛿_C_. Note the different y-axis ranges. A perfectly accurate MAP estimate would have a log-ratio of zero.* ***E)*** *Mean and 95% confidence interval for RMSE of 50 posterior predictions as compared to the synthetic observation for which inference was performed.* ***F)*** *RMSE of posterior prediction generated with MAP parameters as compared to the synthetic observation for which inference was performed.* ***G)*** *Mean and 95% confidence interval for correlation coefficient of 50 posterior predictions compared to the synthetic observation for which inference was performed.* ***H)*** *Correlation coefficient of posterior prediction posterior prediction generated with MAP parameters compared to the synthetic observation for which inference was performed.*

C. Scoping of the method / state of the art

Overall, that there is too much space devoted to comparing these methods and what is best and the manuscript could benefit by placing the inference proposed here in a wider context of numerous similar studies inferring the DFE of beneficial mutations using different experimental settings (eg EMPIRIC or other yeast based method or a plethora of work on E coli and what we have learned about beneficial mutations using these systems).

*We have expanded the review of literature in the introduction. Note that EMPIRIC and other methods have inferred the DFE, but not mutation rates, from experiments with mutant libraries, whereas we have simultaneously inferred mutation rate and fitness effects from experiments that start with an isogenic population. Our inference requires a more complex model that includes the effect of mutation, longer simulations that include the waiting time for appearance of mutations. Moreover, we tackle a harder inference problem due to non-identifiability issues between mutation rate and selection coefficients. Previous papers that applied ABC or similar approaches to similar data from similar experiments have been cited including:*

- Moura de Sousa JA, Campos PRA, Gordo I (2013) An ABC method for estimating the rate and distribution of effects of beneficial mutations. Genome biology and evolution, 5(5):794–806.
- Hegreness M, Shoresh N, Hartl D, Kishony R (2006) An equivalence principle for the incorporation of favorable mutations in asexual populations. Science, 311(5767):1615–1617.
- Barrick, J. E., Kauth, M. R., Strelioff, C. C. & Lenski, R. E. Escherichia coli rpoB mutants have increased evolvability in proportion to their fitness defects. Mol. Biol. Evol. 27, 1338–1347 (2010).

I also think that the ms would be stronger if there is more space devoted to exploring how much the inference is robust to the strong assumptions that all three other nuisance parameters are known without error in advance .

*We have performed sensitivity analysis, and have now extended it to 5 additional parameter combinations (for a total of 10 parameter combinations for “other beneficial mutations”), as shown in Supplementary Figure 14.*

***Supplementary Figure 14. Sensitivity analysis.*** *GAP1 CNV formation rate and selection coefficient inferred using NPE with the Wright-Fisher model does not change considerably when other beneficial mutations have different selection coefficients s_B_ and formation rates 𝛿_B_, except when both s_B_ and 𝛿_B_ are high (purple).*

I hope that the comments I am enclosing along directly on the PDF are also useful

*These comments are indeed useful, thank you! We have made several changes based on comments provided in the pdf including significantly revising the introduction, revising figure legends for greater clarity, and clarifying throughout the number of independent data points per scenario.*

Best regards

Thomas Bataillon
